# Elimination of porcine reproductive and respiratory syndrome virus infection using an inactivated vaccine in combination with a roll-over method in a Hungarian large-scale pig herd

**DOI:** 10.1186/s13028-022-00630-5

**Published:** 2022-05-07

**Authors:** Attila Pertich, Zoltán Barna, Orsolya Makai, János Farkas, Tamás Molnár, Ádám Bálint, István Szabó, Mihály Albert

**Affiliations:** 1 Piginveszt Ltd., Tótkomlós, Hungary; 2 National PRRS Eradication Committee, Budapest, Hungary; 3 National Food Chain Safety Office, Budapest, Hungary; 4 Ceva-Phylaxia Ltd., Budapest, Hungary

**Keywords:** Elimination, Inactivated vaccine, Progressis, PRRS

## Abstract

**Background:**

Porcine reproductive and respiratory syndrome virus (PRRSV) causes severe economic losses worldwide and only four countries in Europe are free from PRRSV. Complete depopulation–repopulation is the safest and fastest, but also the most expensive method for eradicating PRRSV from a population. Another possible way to eliminate an endemic PRRSV infection is to replace the infected breeding stock by gilts reared isolated and protected from PRRSV on an infected farm. With this method it is possible to maintain continuous production on the farm. The authors report the first successful elimination of PRRSV in a Hungarian large-scale pig farm by using an inactivated vaccine and performing segregated rearing of the offspring.

**Case presentation:**

The study was performed on a PRRSV infected farm (Farm A) with 1475 sows. The clinical signs of reproductive failure had been eliminated previously by using an inactivated vaccine (Progressis^®^, Ceva). At the beginning of the elimination programme, gilts intended for breeding were vaccinated at 60 and 90–100 days of age. After that, gilts selected for breeding were vaccinated at 6 months of age, on the 60–70th day of pregnancy and at weaning.

Approximately 1200 piglets from vaccinated sows were transported at 7 weeks of age to a closed, empty farm (Farm B) after being tested negative for PRRSV by a polymerase chain reaction (PCR) method, and then were reared here until 14 weeks of age. At this age, all pigs were tested by PRRS ELISA. Seronegative gilts (n = 901) were subsequently transported from Farm B to a third, closed and empty farm (Farm C), and (having reached the breeding age) they were inseminated here after a second negative serological test (ELISA). At the same time, Farm A was depopulated, cleaned and disinfected. All pregnant gilts were transported from Farm C to Farm A after being re-tested negative for antibodies against PRRSV. Follow-up serology tests were performed after farrowing and results yielded only seronegative animals. Based on the subsequent negative test results, the herd was declared PRRSV free by the competent authority.

**Conclusions:**

The presented farm was the first during the National PRRS Eradication Programme of Hungary to eradicate PRRSV successfully by vaccinating the sows with an inactivated vaccine and performing segregated rearing of the offspring. Production was almost continuous during the whole process of population replacement.

## Background

Porcine reproductive and respiratory syndrome (PRRS) causes significant economic losses in countries with intensive pig production. The estimated yearly loss associated with PRRS is approximately 660 million USD in breeding herds in the USA [1]. Control of PRRS virus (PRRSV) in endemic herds is most commonly achieved by sustaining herd immunity using vaccination [2]. Vaccination can reduce the circulation of the virus in the infected herds, leading to PRRSV stability. The number of viraemic piglets can be significantly reduced and a strong immune response can be achieved with the use of modified live vaccines (MLV) [3–5]. Furthermore, MLV vaccination is able to improve performance parameters in PRRSV endemic farms [6]. However, field experiences revealed safety issues such as reversion to virulence or recombination, and significantly reduced efficacy against field strains not fully homologous to the vaccine strain [7–9]. Killed (inactivated) vaccines (KV) were reported to be safe and can efficiently improve reproductive parameters in PRRSV endemic herds [2, 9, 10]. However, healthy PRRSV carriers may be present among pigs immunised by any PRRSV vaccine [11].

The safest way of eliminating PRRSV from endemic herds is eradication of the infection. This requires a complex approach, careful assessment and planning in areas with high pig density [12]. Depopulation–repopulation is the most efficient and the fastest eradication method, but also the most expensive [4]. PRRSV eradication is a key issue worldwide. In a position statement, the American Association of Swine Veterinarians recommends that the organisation should take on a leading role in the process of PRRSV eradication [13]. Four countries are free from PRRSV in Europe (Norway, Sweden, Finland and Switzerland). Local eradication programmes have started in Denmark and the Netherlands, while eradication was extended to a national level in Scotland in 2018 [14–16].

Eradication of PRRSV in swine herds began in Hungary in 2014 within the framework of a mandatory national programme. Its specifications were laid down in Hungarian national legislation. Results have already been published regarding the eradication of PRRSV infection in backyard pigs and large-scale fattening herds by performing polymerase chain reaction (PCR) and enzyme-linked immunosorbent assay (ELISA) testing followed by regional eradication based on administrative measures (depopulation with state compensation, control of animal movement) [17, 18].

A significant percentage (80–85%) of large-scale breeding herds in Hungary are farrow-to-finish farms [18]. According to scientific literature, infected breeding herds can be stabilised by the use of vaccination and offspring can be kept free of PRRSV until weaning or even to the end of the nursery period [5, 19]. However, if age-segregated production and high levels of internal biosecurity are not rigorously implemented, a PRRSV-free status cannot be maintained in the finishing phase in farrow-to-finish farms [16].

The aim of the present study was to demonstrate how a complex approach was applied in a PRRSV infected Hungarian farrow-to-finish farm, starting with the stabilisation of the sow herd using an inactivated vaccine, followed by strict age-segregated rearing from weaning and eradication by roll-over.

## Case presentation

The study location was a farm (Farm A) with 1250 sows and 225 gilts located in southern Hungary, where replacement gilts were produced internally. The herd was free from brucellosis, leptospirosis and Aujeszky’s disease. The herd became infected with PRRSV in 2008. The identified strain proved to be a European PRRSV 1 strain, showing close genetic relationship with the PRRSV Lelystad reference strain in the ORF5 gene.

**Fig. 1:**
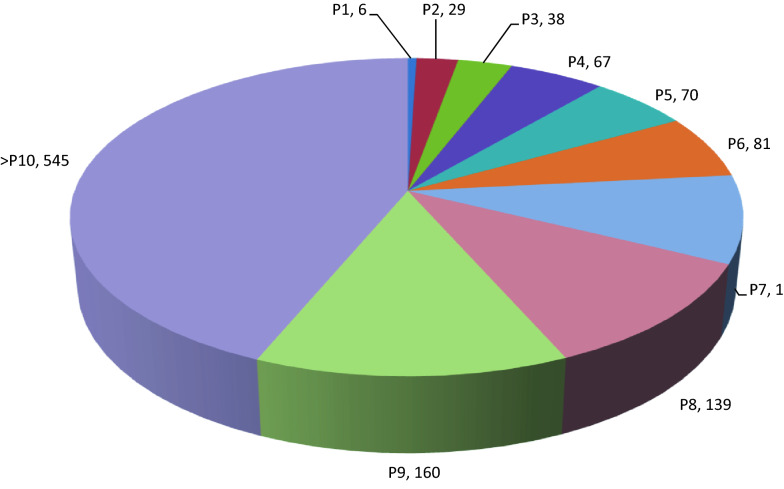
Distribution of sows according to parity (P) in Farm A end of 2014 when the PRRSV eradication programme was in initiated. Numbers in the figure refer to number of sows

Clinical signs of PRRSV infection appeared in the herd in 2008 as a significant number of abortions. In order to mitigate the economic losses, an immunisation program started one month after confirmation of the infection by the use of an inactivated vaccine (Progressis^®^, Ceva). Clinical signs disappeared within 2–3 months after vaccination was initiated and production on the farm stabilised. The Progressis^®^ vaccine was used in breeding animals between 2008 and 2014 according to its specification, but offspring not intended for breeding were not vaccinated. In 2014, at the beginning of the national eradication programme, the age distribution of the sows in the farm was far from the ideal, most of the sows were above parity 7. The production system of the farm was farrow-to-finish from 2008 to 2014, where the finishing phase was discontinued for financial reasons. Piglets were then reared until 70–80 days of age and thereafter sold, but the farm continued to produce replacement gilts. Replacement rate in the breeding herd reached 40% (Fig. 1).

An eradication plan was developed with the involvement of external experts. The PRRSV eradication programme was performed with replacements gilts produced on the farm.

As a first step of the programme, a new immunisation protocol was introduced on the farm. Gilts were vaccinated first at the age of 60 days, followed by a second immunisation at the age of 90–100 days. A third vaccination was performed one month before insemination at the age of 6–6.5 months. The fourth immunisation was applied between 60 and 70 days of pregnancy, and a fifth vaccine was given at weaning. As a result of the regular vaccination, piglets remained PRRSV negative until the age of 90 days as confirmed by PCR tests (Virotype^®^ PRRSV RT-PCR Kit, QIAGEN, Leipzig, Germany). The offspring selected for replacement were transported to two empty premises (Farms B and C) for rearing. Farms B and C were located at a safe distance from farm A and other PRRSV endemic farms. (Distance between farms: A to B approx. 6 km, B to C approx. 20 km; C to A approx. 16 km).

Piglets that were PRRSV negative by PCR testing at the age of 7 weeks were kept in a separate, closed unit (Farm B) until the age of 14 weeks and handled by personnel working strictly on Farm B. Piglets were transported from Farm A in batches of approximately 300 piglets. Before being transported to farm B, the selected group of animals were sampled for PCR tests to achieve a 95% level of confidence and account for 5% prevalence (Table 1). PCR analyses of sera were performed at the Hungarian National PRRS Reference Laboratory (INgezim PRRS Universal ELISA, Eurofins Technologies).

**Table 1:** Overview of animal numbers on the individual farms and testing strategy

Farm	Age	Number of animals in the population	Gilt replacement	Sample number	Diagnostic examination	Remarks
A	7 weeks	18,205	1200	1140	PCR	PRRS infected herd
B	14 weeks	901	901	901	ELISA	
C	23 weeks	901	855	855	ELISA	
C	37 weeks	855	855	855	ELISA	
A	9 months	855	855	855	ELISA	Depopulated, disinfected farm
A	15 months	855	855	855	ELISA	Disinfected farm
A	21 months	855	–	855	ELISA	Qualifying examination
A + B + C				6316		

All the gilts reared in Farm B were serologically tested for antibodies against PRRSV at the age of approximately 14 weeks and yielded negative results. Serology tests were performed at the Hungarian National PRRS Reference Laboratory. The seronegative animals were transported to a third, closed empty farm (Farm C) and kept according to quarantine rules. The pens and the farm were cleaned and disinfected between groups of animals. On the 60th day after arrival at Farm C, a second serological testing was carried out on all gilts. When reaching breeding age, PRRSV seronegative gilts were inseminated at Farm C, and kept there until farrowing. Personnel designated to Farm C worked only on Farm C and they were not allowed be in contact with any other pigs or enter other premises. Feed for Farm C was supplied from a separate feed mill. Farm B had to be used in the elimination programme because the capacity of the quarantine facility on Farm C was not enough for 300 piglets.

Insemination was stopped on Farm A and all animals were slaughtered after the last farrowing. The empty Farm A was subsequently cleaned and disinfected in a time frame of about 3 months. Farm A was repopulated from Farm C by pregnant gilts free of PRRSV infection. Before transport to Farm A, all pregnant gilts in Farm C were serologically tested for antibodies against PRRSV, and seronegative gilts were moved to Farm A, where farrowing took place. Herd classification of the new population in Farm A was performed by testing all sows serologically twice with a six-month interval. Since all tests yielded negative results, the competent authority declared the farm free from PRRSV in 2019. The free status of the farm is still maintained (17 February 2022).

## Discussion and conclusions

PRRSV was successfully eliminated in a Hungarian large-scale farrow-to-finish pig herd where herd replacement was achieved by applying the roll-over method with the use of an inactivated vaccine. Gilts were born and reared on the infected farm but under PRRSV-free conditions, then segregated and reared on a separate farm. A mandatory PRRSV eradication programme applicable to all Hungarian swine farms was launched in 2014. When evaluating the PRRSV status in Hungary, it turned out that most large-scale pig farms follow a farrow-to-finish strategy. The all-in-all-out procedure was not carried out consequently during the production phases in several infected herds, which lead to PRRSV persistence and circulation [18].

Stricker [20] pointed out that it is not possible to reach PRRSV free status if animals are retained in the different production phases. The most plausible, but at the same time the most expensive method of PRRSV eradication, is depopulation-repopulation. But the management of Hungarian breeding herds wanted to achieve eradication on the PRRSV endemic farms with the lowest possible costs and economic losses.

It is crucial to stabilise the sow herd and to establish the stable PRRSV status on an infected farm before starting an eradication programme [5, 21]. A stable PRRSV status in an endemic herd can be achieved by vaccination, while determining a PRRSV stable status of a herd requires regular laboratory testing (PCR, ELISA, sequencing). Moreover, high levels of both external and internal biosecurity must also be ensured in order to achieve a stable status of the herd [22]. Analysis of data in PRRSV infected herds has shown that a PRRSV stable status can be achieved in an average of 26.6 weeks after starting vaccination using an MLV. However, PRRSV can still be detected but at a low viral load and with low frequency [22].

By utilising the above applied principles and practical experience, several countries performed successful local and regional eradication with the use of MLVs and biosecurity measures [19, 23]. PRRSV eradication in the Hungarian herd was carried out in a complex way, applying strict biosecurity measures and vaccination. Immunisation with inactivated vaccines has previously been recommended on PRRSV endemic farms as a therapeutic tool [2]. Although some publications mention that immune response is relatively weak after using an inactivated vaccine [24], a strong protection was shown to develop when vaccinating previously infected animals [2, 9]. Papatsiros et al. [10] reported a similar experience in a farrow-to-finish herd, where an inactivated vaccine was used for 2 years and lead to significant improvement in reproductive parameters. However, despite vaccination, PRRSV was present in the finishing unit. This phenomenon was observed in the Hungarian herd as well.

For successful elimination of PRRSV in farrow-to-finish type farms it seems necessary to break the chain of infection between the nurseries and finishing in at least one production cycle, and to perform regular laboratory testing to check if PRRSV free status is maintained in finishers [18, 25].

In the Hungarian farm, the sow herd was stabilised by applying an inactivated vaccine between 2008 and 2014. With this strategy, piglets could maintain a PCR-negative and seronegative PRRSV status throughout the nursery phase (until 80–90 days of age). However, nearly 100% became seropositive within 3–4 weeks after arrival at the finishing unit, which indicate persistent PRRSV circulation.

The results confirm the conclusion of Linhares [22] that it is possible to successfully implement a vaccination and biosecurity programme even in heavily infected farms to guarantee PRRSV free status until the end of the nursery phase.

Not only vaccination, but also an increased gilt replacement up to 40% was implemented in the aged sow herd of farm A. The higher replacement rate and culling of the old sows in the closed, vaccinated breeding population probably reduced the prevalence of PRRSV infection. It has previously been shown that reproductive and litter parameters in sows are improved after the first dose of an inactivated vaccine [10]. It is known that performance of breeding sows gradually improves with their parity [26]. These are the main reasons why offspring of older sows (> parity 3) were selected for herd replacement at the start of the repopulation process.

Laboratory examinations are key elements in controlling the progress of an eradication programme. For the purpose of PRRSV monitoring, PCR and ELISA tests should be performed regularly, at defined time points and contribute to the validation of the progress of the PRRSV eradication [23].

When designing the schedule and determining the number of samples for laboratory testing, the main goal is to be able to identify infected individuals in time and with high probability.

Toman et al. [19] reported successful eradication with the use of an MLV, but sequencing had to be performed from PCR positive animals. In our case, sequencing was not necessary because an inactivated vaccine was used, which reduced the costs. Once the animals were tested negative with PCR, serological tests were sufficient for the control of the replacement gilt population reared on the closed farms B and C. ELISA tests were always performed before transports and at the end of the quarantine period. The last two serological tests on Farm A were performed in order to declare a PRRSV free status.

The most important technical measures leading to the successful acquisition of the PRRSV free status on the farm were regular vaccination and increased culling rate of older sows that contributed to the establishment of a PRRSV stable sow herd. Further measures also contributed to the success, such as discontinuing fattening in the infected farm, as well as restricting transportation and breeding to animals with negative laboratory test results during the eradication process. By applying these steps and implementing strict internal and external biosecurity measures it was possible to achieve and maintain a PRRSV free herd status throughout every phase of production.

The economic advantage of the above mentioned PRRSV elimination programme was that except for a minimal, 3-month-long interruption, production could be maintained on the infected farm throughout a successful PRRSV eradication process.

## Data Availability

Data of the farm, as well as diagnostic PCR and ELISA results are available from AP (Mail: attila.pertics@gmail.com).
